# Eating behavior and body composition across childhood: a prospective cohort study

**DOI:** 10.1186/s12966-018-0725-x

**Published:** 2018-10-01

**Authors:** Ivonne P. M. Derks, Eric J. G. Sijbrands, Melissa Wake, Farah Qureshi, Jan van der Ende, Manon H. J. Hillegers, Vincent W. V. Jaddoe, Henning Tiemeier, Pauline W. Jansen

**Affiliations:** 1 Department of Child and Adolescent Psychiatry/ Psychology, Erasmus MC-University Medical Center, Wytemaweg 80, 3000 CA Rotterdam, the Netherlands; 2 The Generation R Study Group, Erasmus MC-University Medical Center, Rotterdam, the Netherlands; 3 Department of Internal Medicine, Erasmus MC-University Medical Center, Rotterdam, the Netherlands; 4 Murdoch Children’s Research Institute, Parkville, Australia; 5 Department of Pediatrics, The University of Melbourne, Parkville, Australia; 6 Department of Social and Behavioral Sciences, Harvard TH Chan School of Public Health, Boston, MA USA; 7 Department of Epidemiology, Erasmus MC-University Medical Center, Rotterdam, the Netherlands; 8 Department of Pediatrics, Erasmus MC-University Medical Center, Rotterdam, the Netherlands; 9 Department of Psychology, Education, and Child Studies, Erasmus University Rotterdam, Rotterdam, the Netherlands

**Keywords:** Eating behaviors, Appetite, BMI, Fat mass, Adiposity, Longitudinal, Cohort

## Abstract

**Background:**

Although many cross-sectional studies reported that children with overweight or obesity show more food approaching and less food avoidant eating behaviors, there is a lack of replication in longitudinal studies. Therefore, the question remains whether healthcare professionals should target eating behaviors in childhood obesity interventions and prevention. We aimed to examine the longitudinal and possible bi-directional associations between eating behavior and body composition across childhood.

**Methods:**

Data was included from 3331 children participating in the Generation R Study. At 4 and 10 years, mothers reported on the Child Eating Behavior Questionnaire including the subscales Food Responsiveness, Enjoyment of Food, Emotional Overeating and Satiety Responsiveness, and children’s BMI was measured. Body composition, consisting of Fat Mass Index and Fat Free Mass Index was measured at 6 and 10 years with Dual-energy-X-ray-Absorptiometry scans.

**Results:**

Cross-lagged models including both directions of the BMI – eating behavior association showed that a higher BMI at the age of 4 years predicted more food responsiveness and enjoyment of food and less satiety responsiveness at 10 years (e.g. satiety responsiveness:β = − 0.10, 95% CI = − 0.14, − 0.07), but no associations were found in the opposite direction. For emotional overeating, however, a bi-directional association was found with BMI predicting more emotional eating and vice versa. Multivariable linear regression analyses showed that associations were stronger for Fat Mass Index than for Fat Free Mass Index.

**Conclusions:**

Results showed that a higher BMI, and particularly higher fat mass, at pre-school age predicted more food approaching and less food avoidant eating behaviors at the age of 10 years, rather than the hypothesized reverse direction. This suggests that increased adiposity in early childhood might upregulate appetite and related eating behaviors.

**Electronic supplementary material:**

The online version of this article (10.1186/s12966-018-0725-x) contains supplementary material, which is available to authorized users.

## Background

Eating behaviors and Body Mass Index (BMI) are closely linked across the lifespan [1]. Numerous cross-sectional studies in childhood reported that children with a higher weight status show more food approaching and less food avoidant eating behaviors. Namely, children with overweight or obesity are more sensitive to external food cues (i.e. food responsiveness), they tend to eat more when they experience negative emotions (i.e. emotional overeating) and show more pleasure and interest in eating (i.e. enjoyment of food) than healthy weight children, which are considered food approaching behaviors. In turn, children with overweight or obesity showed to be less responsive to their internal satiety cues (i.e. satiety responsiveness) than their healthy weight counterparts, where satiety responsiveness is considered a food avoidant behavior [2–14]. Two of these studies showed a gradient relationship of these behaviors across the BMI span, indicating a dose-response relationship between BMI and eating behaviors [3, 7]. In contrast to this abundance of evidence, two studies found no cross-sectional assocation between eating behaviors and weight status in children [15, 16]. Consequently, scholars and policy makers have advocated that eating behaviors should be targeted in childhood obesity interventions [17]. This implication is, however, hardly supported by prospective research. Only two studies showed that higher food responsiveness and a poor satiety responsiveness at 3 months of age, but not at 12 months, were associated with subsequent BMI until 15 months of age [18, 19]. In contrast, Mallan et al. (2014) reported a prospective association of poorer satiety responsiveness at 2 years with a higher BMI at 4 years of age, but found no association between food approaching behaviors and BMI [20]. Besides these few studies in early life with a relative short follow-up period, the evidence for the potential effect of eating behavior on subsequent BMI is rather poor. Conversely, a higher BMI might also affect eating behavior through an increase in energy needs and up-regulation of appetite. A recent study conducted among 807 Norwegian community children found evidence for this, as higher fat mass levels at age 6 predicted an increase in food responsiveness by age 10 years, while more muscle mass predicted a decrease in satiety responsiveness [21]. However, the association in the opposite direction – examining whether eating behavior affects later body composition – was not reported.

Clearly, prospective studies with repeated measurements are needed to ascertain potential (reverse) causality in the eating behavior – BMI association. To date, only two longitudinal studies investigated both directions during one period in separate models and found evidence of bi-directionality in the relationship between food responsiveness and BMI, while results on satiety responsiveness were inconsistent [22, 23]. Moreover, other eating behaviors such as emotional overeating and enjoyment of food, as well as fat- and fat free mass were not examined. In order to determine the strongest direction of effect, both directions of the association between eating behavior and BMI must be examined in a one model. Determining causes and consequences will help identify whether eating behaviors are indeed a right target for obesity interventions aimed at primary prevention, which is of upmost importance for policy makers. Educating parents on the development of eating behaviors and how to change unhealthy eating habits is only effective when eating behaviors indeed have a long-term effect on weight development. If it appears that food approaching and food avoidant eating behaviors are a consequence of a high weight status, these preventive interventions may not be effective in reducing childhood obesity. Therefore, the aim of this study was to prospectively examine both directions of the association between eating behavior and body composition across childhood (from 4 and 6 to 10 years) in a large population-based cohort in the Netherlands. We expect that more food responsiveness, emotional overeating, enjoyment of food and less satiety responsiveness prospectively predict a higher BMI - and particularly a higher fat mass - later in childhood, and that the opposing direction will be weaker.

## Methods

### Study design and population

This study was embedded in the Generation R Study, a population-based prospective birth cohort situated in Rotterdam, the Netherlands [24]. All pregnant women living in Rotterdam with an expected delivery date between April 2002 and January 2006 were invited (participation rate: 61%). Written informed consent was obtained from all participants and the Medical Ethical Committee of the Erasmus Medical Center approved the study. Full consent for the postnatal phase was obtained for 7294 children and their parents (73,7% of those originally enrolled). Information on eating behavior at both 4 and 10 years was available for 3514 children. The final study sample consisted of 3331 children, for whom BMI was also assessed at the age of 10 years (Additional file 1: Figure S1). The study sample for analyses with body composition at 6 years was slightly smaller (*n* = 3097), due to missing body composition data. Missing values on BMI at 4 years (*n* = 1136) were handled with missing imputation procedures (see Statistical analyses). Children included in this study were more often girls and had more often a Dutch background (*n* = 3331), compared to children who were excluded due to missing data (*n* = 3963). Further, children included in this study had mothers with a lower median BMI and came from families with a higher household income (all *p*-values< 0.01).

### Measures

#### Child eating behavior

Eating behavior was assessed twice using the same measure, when children were 4 and 10 years old. At both time points, mothers reported on their children’s eating behavior with the Child Eating Behavior Questionnaire (CEBQ) [25]. The CEBQ is a 35-item instrument that assesses variation in eating behaviors among children. For this study, three subscales were included with high scores reflecting food approaching behavior, namely: emotional overeating, food responsiveness and enjoyment of food, as well as the subscale satiety responsiveness reflecting food avoidant behavior. Emotional overeating consists of 4 items (e.g. “My child eats more when he/she is upset”), food responsiveness is a 5- item subscale which assesses children’s sensitivity to external food cues (e.g. “Given the choice, my child would eat most of the time”) and enjoyment of food is a 4-item subscale (e.g. “My child loves food”). Satiety responsiveness consists of 9 items combining the subscales satiety responsiveness and slowness in eating - considered as a response to the progressive triggering of internal satiety cues during food intake [26]. This combined scale has been validated against behavioral tests of food intake [3, 27]. The CEBQ has well-established psychometric properties, including good test-retest reliability, internal consistency and concurrent validity with actual/observed eating behavior [4, 12, 25, 27]. At both time points, the subscales showed good internal consistency in The Generation R sample, with Cronbach’s alpha at the age of 4 years ranging from 0.78 to 0.89, and at 10 years ranging from 0.84 to 0.92.

#### Child BMI and body composition

At 4 years, children’s growth characteristics were obtained as part of routine health care by trained staff of the community Child Health Centers, where growth and health are regularly monitored for all children living in the Netherlands. At 10 years, children visited the dedicated Generation R research center in Rotterdam. At both occasions, child height was measured in standing position using a Harpenden stadiometer and weight was measured without shoes and heavy clothing using a mechanical personal scale (SECA). Sex- and age- adjusted BMI (kg/m^2^) SD scores were calculated according to the Dutch reference growth curves (https://growthanalyser.org/) [28].

During the in-person visits at the ages of 6 and 10 years, a detailed assessment of body composition took place by using Dual-energy-X-ray-Absorptiometry (DXA) scan (iDXA, GE-Lunar, 2008, Madison, WI, USA). Body fat mass and fat free mass were measured while children were laying down in horizontal position. Sex- and age adjusted Fat Mass Index (FMI, fat mass (kg)/length (m)^2^) and Fat Free Mass Index (FFMI, fat free mass (kg)/length(m)^2^) SD scores were calculated, based on all participating children with body composition available, for each time point separately.

#### Covariates

Several covariates were considered as potential confounders in the association between eating behavior and body composition. Information on child sex and birth weight were obtained from midwife- and hospital registries. Birth weight was transformed into standardized scores adjusted for gestational age according to the Swedish reference standards [29]. Ethnicity of the child was based on the country of birth of both parents, which was assessed by prenatal questionnaires, as well as maternal highest attained educational level. After birth, mothers reported on the duration of breastfeeding by postal questionnaire when the child was 2 months, 6 months and 12 months old. Mothers were asked whether they ever breastfed their child, and if yes, duration of any breastfeeding was assessed by asking at what age of the infant they stopped breastfeeding (in months). Depression and anxiety symptoms of the mother were assessed with the validated Brief Symptom Inventory (BSI) when children were 3 years old [30]. The BSI consists of 53 items about how the participant felt during the last seven days with answering options on a five-point scale, ranging from “0 = not at all” to “4 = extremely”. The Depression and Anxiety scales both consists of six items, for example “Not interested in anything anymore” for depression and “Having fear or panic attacks” for anxiety. Mean scale scores were calculated for both scales separately. At the 6 years visit, maternal BMI was measured at the research center and in the same examination household income was assessed by postal questionnaire.

### Statistical analyses

We used multiple imputation (i.e., fully conditional specification) to impute missing data on covariates and child BMI at the age of 4 years. We analyzed 20 imputed datasets of which the results were pooled. All study variables and additional information (i.e. BMI at 12 other time points from birth to 10 years) were included in the imputation model. For the cross-lagged modeling, Full Information Maximum Likelihood estimation was used to deal with missing data.

Sum scores of CEBQ subscales were standardized for comparison purposes. Potential confounders were included in the analyses when they changed one of the unadjusted effect estimates in the eating behavior-BMI association by more than 5% but did not have to be significant predictors of the outcome. As a result, the following confounders were included: child ethnicity, birth weight, maternal educational level, maternal BMI, maternal anxiety symptoms and household income. We examined the association between eating behaviors and body composition with a step-by-step approach including cross-sectional and uni-directional relationships, which enhances the comparability with previous studies. First, cross-sectional associations between child BMI and body composition at 10 years were studied using multivariable linear regression analyses, adjusted for confounders. Next, using multivariable linear regression, we studied the longitudinal relationship between eating behaviors at the age of 4 years with BMI, FMI and FFMI SD scores at 10 years, adjusted for confounders (model 1), and additionally adjusted for BMI SD score at 4 years to assess the temporal relationship adjusted for baseline (model 2). Associations in the opposing direction were also examined with multivariable linear regression analyses. In model 1, the association of BMI SD score at 4 years, FMI and FFMI SD scores at 6 years with subsequent eating behaviors at the age of 10 years were examined adjusted for confounders. In model 2, we additionally adjusted for the corresponding eating behavior at the age of 4 years for temporal purposes. We previously reported on cross-sectional associations between eating behavior and BMI at 4 years [5].

Finally, to better account for the complexity of the data, the directionality of the association between child eating behavior and BMI was examined with a cross-lagged modeling approach for each subscale of the CEBQ separately. In this type of analysis, the opposing prospective associations (the lagged effects) between eating behavior and BMI are studied in the same model while accounting for cross-sectional relations and for continuity between BMI assessments and between eating behavior assessments over time. Confounders were regressed on the BMI and eating behavior assessments at age 4 years. Wald tests were used to determine the significance of differences between the lagged coefficients for each model. Multivariable linear regression analyses were performed with SPSS version 24.0 (IBM Corp.), and the cross-lagged analyses were performed with Mplus, version 7.11 (Muthèn & Muthèn).

## Results

Non-imputed sample characteristics are presented in Table 1. Children had mostly a Dutch background (71.5%), and mothers had a mean BMI of 23.9 (SD = 5.1). At 10 years, emotional overeating, food responsiveness and enjoyment of food were cross-sectionally associated with BMI, FMI and FFMI SD scores (e.g. food responsiveness with FMI SD score: B = 0.31, 95%CI = 0.28, 0.33), whereas satiety responsiveness was negatively associated with these body composition measures (e.g. satiety responsiveness with FFMI: B = − 0.27, 95%CI = − 0.30, − 0.24) (Table 2).

**Table 1:** Descriptive characteristics of the study sample

Sample characteristics	Total n	No. (%), mean (SD) or median (IQR)^1^
Age at 10 years visit, mean (SD)	3331	9.8 (0.3)
Sex, No. % boys	3331	1622 (48.7)
Child ethnicity, No. %	3324	
Dutch		2377 (71.5)
Other Western		307 (9.2)
Non-Western		640 (19.2)
Birth weight in grams, mean (SD)	3329	3447.7 (566.4)
Birth weight for gestational age, mean (SD)	3317	−0.01 (1.01)
BMI at age 4 years, mean (SD)	2195	15.8 (1.3)
BMI at age 10 years, mean (SD)	3331	17.2 (2.4)
Maternal education level, No. %	3213	
Low (no education - high school)		332 (10.3)
Medium (Lower vocational education)		885 (27.5)
High (Higher vocational education and university)		1996 (62.1)
Maternal BMI, median (IQR)	3142	23.9 (5.1)
Maternal anxiety symptoms, median (IQR)^2^	3040	0.00 (0.17)
Household income, No. %	3037	
Low (< 1600 euro per month)		268 (8.8)
Medium (1600–4000 euro per month)		1470 (48.4)
High (> 4000 euro per month)		1299 (42.8)

^1^Values are percentages for categorical variables, means (standard deviations) for continuous normally distributed variables and medians (interquartile ranges) for continuous, non-normally distributed variables and all values are based on original data. ^2^ Maternal psychopathology symptoms were assessed with the Brief Symptom Inventory

Abbreviations: *BMI* Body mass index, *IQR* Inter quartile range, *SD* Standard deviation

**Table 2:** Cross-sectional associations between eating behaviors and body composition at the age of 10 years

CEBQ subscales (z-scores)	Body composition		
	BMI SD score B (95% CI)	FMI SD score B (95% CI)	FFMI SD score B (95% CI)
Emotional overeating	0.13 (0.10, 0.16)	0.12 (0.10, 0.15)	0.07 (0.04, 0.10)
Food responsiveness	0.35 (0.32, 0.38)	0.31 (0.28, 0.33)	0.23 (0.20, 0.26)
Enjoyment of food	0.21 (0.17, 0.24)	0.15 (0.12, 0.17)	0.17 (0.14, 0.20)
Satiety responsiveness	−0.28 (− 0.31, − 0.25)	− 0.16 (− 0.19, − 0.13)	−0.27 (− 0.30, − 0.24)

Values are linear regression coefficients, the CEBQ subscales were transformed into z-scores. All values were significant at *p* < 0.001. Analyses were adjusted for child ethnicity, birth weight for gestational age SD score, household income, maternal educational level, maternal BMI and maternal anxiety symptoms. *N* = 3331

Abbreviations: *CEBQ* Child eating behavior questionnaire, *BMI* Body mass index, *FMI* Fat mass index, *FFMI* Fat free mass index, *SD score*, Standard deviation score

Next, longitudinal associations between eating behavior at age 4 years with BMI and body composition at 10 years were examined (Table 3). Adjusted for covariates and baseline BMI, emotional overeating at age 4 years was positively associated with a higher BMI and FMI 6 years later (e.g. B = 0.03, 95%CI = 0.00, 0.06). Food responsiveness was not associated with later BMI or body composition measures. Enjoyment of food showed a negative association with FMI (B = − 0.03, 95%CI = − 0.05, − 0.00) and a positive association with FFMI (B = 0.05, 95%CI = 0.02, 0.08) at 10 years. Satiety responsiveness predicted lower FFMI SD scores 6 years later (B = − 0.08, 95%CI = − 0.11, − 0.05), but not BMI or FMI SD score.

**Table 3:** Longitudinal associations between eating behavior at 4 years and body composition at the age of 10 years

CEBQ subscales at age 4 years (z-scores)		Body composition at age 10 years		
		BMI SD score B (95% CI)	FMI SD score B (95% CI)	FFMI SD score B (95% CI)
Emotional overeating	Model 1	0.03 (0.00, 0.06)	0.03 (0.00, 0.06)*	0.01 (− 0.03, 0.04)
	Model 2	0.03 (0.00, 0.06)*	0.03 (0.00, 0.06)*	0.01 (−0.02, 0.03)
Food responsiveness	Model 1	0.14 (0.10, 0.17)**	0.09 (0.06, 0.12)**	0.13 (0.10, 0.16)**
	Model 2	0.02 (−0.01, 0.05)	0.02 (−0.01, 0.04)	0.02 (− 0.01, 0.05)
Enjoyment of food	Model 1	0.09 (0.06, 0.12)**	0.03 (0.00, 0.06)*	0.13 (0.10, 0.16)**
	Model 2	0.00 (−0.03, 0.03)	−0.03 (− 0.05, − 0.00)*	0.05 (0.02, 0.08)*
Satiety responsiveness	Model 1	− 0.15 (− 0.19, − 0.12)**	−0.06 (− 0.09, − 0.03)**	−0.19 (− 0.22, − 0.16)**
	Model 2	−0.03 (− 0.06, 0.00)	0.02 (− 0.01, 0.05)	−0.08 (− 0.11, − 0.05)**

Values are linear regression coefficients. Model 1 was adjusted for child ethnicity, birth weight for gestational age SD score, household income, maternal educational level, maternal BMI and maternal anxiety symptoms. Model 2 was additionally adjusted for BMI at 4 years. N = 3331. * represents p-value < 0.05. ** represents *p*-value < 0.001

Abbreviations: *CEBQ* Child eating behavior questionnaire, *BMI* Body mass index, *FMI* Fat mass index, *FFMI* Fat free mass index, *SD score* Standard Deviation score

Associations in the opposing direction, from BMI at 4 years and body composition at 6 years to eating behaviors at 10 years, are presented in Table 4. A higher BMI predicted more emotional overeating, food responsiveness and enjoyment of food, and less satiety responsiveness 6 years later, adjusted for covariates and baseline eating behavior (e.g. emotional overeating: B = 0.09, 95%CI = 0.05, 0.12). The same pattern was also found for body composition measures at the age of 6 years and eating behaviors 4 years later. Adjusted for confounders and corresponding eating behaviors at 4 years (model 2), higher FMI and FFMI at 6 years were associated with more emotional overeating, food responsiveness and enjoyment of food, and less satiety responsiveness at 10 years (e.g. FMI with satiety responsiveness: B = − 0.19, 95%CI = − 0.23, − 0.15). Overall, higher effect estimates for FMI (i.e. food responsiveness: B = 0.37, 95%CI = 0.32, 0.41) than for FFMI (B = 0.17, 95%CI = 0.13, 0.20) were observed according to non-overlapping confidence intervals.

**Table 4:** Longitudinal associations between body composition at 4 and 6 years and eating behavior at the age of 10 years

Body composition		CEBQ subscales at age 10 years (z-scores)			
		Emotional overeating B (95% CI)	Food responsiveness B (95% CI)	Enjoyment of food B (95% CI)	Satiety responsiveness B (95% CI)
At age 4 years					
BMI SD score	Model 1	0.09 (0.05, 0.12)**	0.23 (0.19, 0.28)**	0.17 (0.13, 0.21)**	−0.24 (− 0.28, − 0.20)**
	Model 2	0.09 (0.05, 0.12)**	0.15 (0.11, 0.19)**	0.09 (0.06, 0.13)**	−0.12 (− 0.16, − 0.08)**
At age 6 years (n = 3097)					
FMI SD score	Model 1	0.17 (0.12, 0.21)**	0.45 (0.40, 0.49)**	0.23 (0.18, 0.27)**	−0.26 (− 0.31, − 0.22)**
	Model 2	0.16 (0.11, 0.20)**	0.37 (0.32, 0.41)**	0.17 (0.13, 0.21)**	−0.19 (− 0.23, − 0.15)**
FFMI SD score	Model 1	0.06 (0.02, 0.10)*	0.22 (0.19, 0.26)**	0.17 (0.13, 0.20)**	−0.28 (− 0.32, − 0.24)**
	Model 2	0.06 (0.03, 0.10)*	0.17 (0.13, 0.20)**	0.10 (0.06, 0.13)**	−0.17 (− 0.21, − 0.14)**

Values are linear regression coefficients. Model 1 was adjusted for child ethnicity, birth weight for gestational age SD score, household income, maternal educational level, maternal BMI and maternal anxiety symptoms. Model 2 was additionally adjusted for the corresponding eating behavior subscale at 4 years. *N* = 3331, for the analyses with FMI and FFMI at age 6 years, *n* = 3097. * represents p-value < 0.05. ** represents p-value < 0.001

Abbreviations: *CEBQ* Child eating behavior questionnaire, *BMI* Body mass index, *FMI* Fat mass index, *FFMI* Fat free mass index, *SD score* Standard Deviation score

**Fig. 1:**
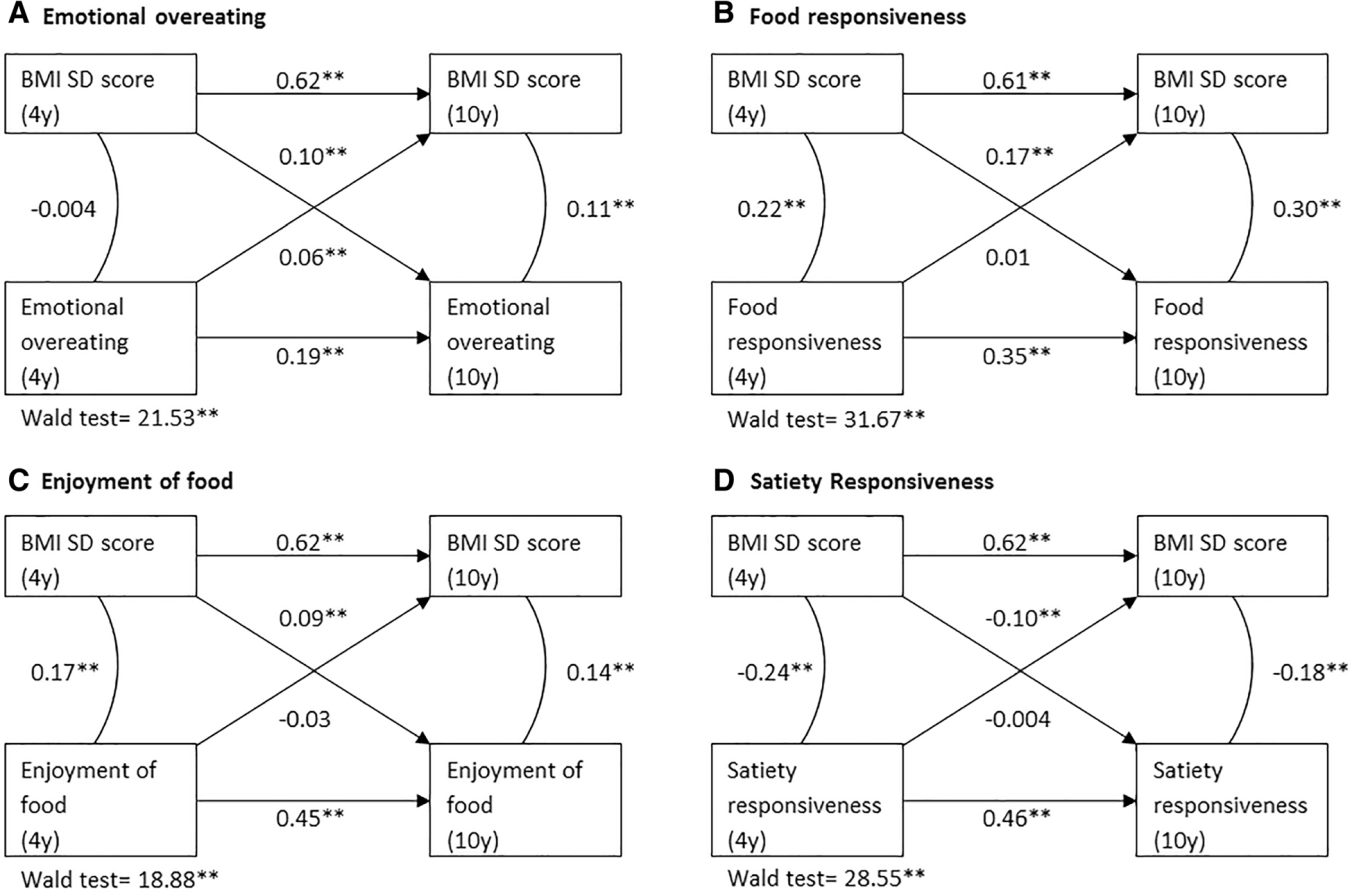
Cross-lagged models of BMI with emotional overeating (**a**), food responsiveness (**b**), enjoyment of food (**c**) and satiety responsiveness (**d**) across childhood, *n* = 3331. Values represent standardized linear regression coefficients (Betas). Models are adjusted for child ethnicity, birth weight for gestational age SD score, household income, maternal educational level, maternal BMI and maternal anxiety symptoms. Wald tests compare the paths from eating behavior at 4 years to BMI at 10 years versus BMI at 4 years to eating behavior at 10 years, for which a significant Wald test indicates a significantly stronger pathway. Fit indexes for each model were: Comparative Fit Index ≥0.790, Root Mean Square Error Of Approximation ≤0.083. * represents *p*-value < 0.05. ** represents *p*-value < 0.001

Results of the cross-lagged models are shown in Fig. 1. Although the model fit indices, especially the CFI and TLI, were poor, the parameter estimates were very reasonable, suggesting that the models are consistent with the data [31]. Results indicated that, when accounting for cross-sectional associations and continuity over time, a higher BMI at age 4 predicted more food responsiveness and enjoyment of food and less satiety responsiveness at 10 years (e.g. satiety responsiveness: β = − 0.10, 95%CI = − 0.14, − 0.07). We found no associations in the opposite direction, from eating behavior to subsequent BMI. A higher BMI predicted subsequent emotional overeating (β = 0.10, 95%CI = 0.06, 0.14) and higher levels of emotional overeating also predicted more weight gain over time (β = 0.06, 95%CI = 0.03, 0.09). For all four eating behavior subscales, the pathway from BMI to eating behavior was stronger than the reversed, as indicated by significant Wald tests.

## Discussion

To the author’s knowledge, this study was the first to test the directionality of the association between eating behavior and BMI across childhood by using a cross-lagged modeling approach. With this method, we showed that more food approaching and less food avoidant eating behaviors were mainly a consequence of a high BMI in childhood, rather than the reverse direction. For emotional overeating, a bi-directional relationship was found, indicating that emotional overeating was both a predictor and a consequence of a relatively high BMI. In line with our findings regarding BMI, we found no clear evidence for eating behaviors at 4 years predicting fat mass or fat free mass six years later, but instead, higher fat mass and fat free mass at the age of 6 years were both associated with subsequent eating behaviors. Although effect sizes were somewhat stronger for fat mass than for fat free mass, we did not observe distinct effects on eating behaviors. The findings of this study contradict our a priori hypothesis that more food approaching and less food avoidant eating behaviors would prospectively predict a higher BMI and fat mass.

Previous studies showed that eating behavior prospectively predicted weight gain (mostly by examining delta weight or BMI, e.g. BMI outcome - BMI baseline) [18–20, 22, 23], while we found limited evidence of a prospective association after adjusting for baseline BMI. Moreover, in the cross-lagged models, the effect estimates of food responsiveness, enjoyment of food and satiety responsiveness on BMI in later childhood were further attenuated. Our specific type of modelling might explain differences in conclusions between this study and other studies, as previous studies did not examine the bi-directional nature of eating behaviors and BMI in a cross-lagged model. Another possibility for the inconsistency in findings would be that most previous studies were conducted before 4 years of age, which is the baseline age of our study. Possibly, the association between eating behavior and weight gain differs across the childhood years, with infancy and early childhood reflecting a critical period for influences of appetite on weight development. Future studies with repeated measurements of eating behavior and BMI from early infancy to late childhood are needed to examine this hypothesis.

An important finding is that a higher BMI in the pre-school years predicted more food responsiveness and enjoyment of food, and less satiety responsiveness at the age of 10 years. Two previous studies also found an association in this direction, in infancy and mid-childhood [22, 23]. Moreover, Steinsbekk et al. (2017) showed that fat mass and muscle mass had distinct associations with specific eating behaviors over time: higher fat mass at 6 years and 8 years was only associated with more food responsiveness at 8 and 10 years, respectively, while in turn, higher muscle mass was only associated with less satiety responsiveness [21]. This is partly replicated in our study; although we did not find distinct associations for fat mass and fat free mas, children with higher fat mass were more likely to eat in response to external food cues, while those with higher fat free mass were more likely to eat even when they were full. Related literature also found evidence for a potentially causal relationship between BMI and other dimensions of eating behavior. A recent Mendelian randomization study showed that a higher BMI in childhood predicted more disordered eating such as binge eating in adolescence [32]. Our study adds to this previous work by showing that a higher BMI at 4 years of age already was associated with eating behaviors a few years later. The reported standardized effect sizes of the cross-lagged models were small, but comparable to those reported in previous studies on the relationship between BMI and subsequent eating behaviors [22, 23].

Biological mechanisms linking a high weight status and subsequent eating behaviors may potentially explain our findings. First, an increased BMI may up-regulate appetite through an increased energy-balanced set point at which the body tries to maintain the current weight status. Obesity can be considered as a condition of body energy regulation at an elevated set point, for which more energy intake is needed [33, 34]. Secondly, overeating might be a result of decreased leptin sensitivity. The satiety-hormone leptin has an inhibitory effect on appetite regulation in healthy-weight individuals. However, in obese adults chronically elevated leptin levels are causing impaired leptin-signaling capacity in the hypothalamus, leading to leptin resistance [35, 36]. Likewise, a strong positive correlation between leptin concentrations and fat mass has been observed in children with obesity [37, 38]. Potentially, subclinical levels of excess weight might already affect children’s leptin sensitivity, which consequently decreases satiety levels. Thirdly, dopamine - a neurotransmitter involved in food intake regulation by modulating food-reward sensitivity - might also play a role in unhealthy eating behavior among children with excess weight. In individuals with obesity, availability of dopamine receptors in the brain striatum is decreased, resulting in difficulty to obtain feelings of reward from food. As a result, more food is needed to obtain the same rewarding feeling [39, 40]. To date, these hypothetical biological mechanisms have been examined primarily among obese adults, but it is unclear whether these mechanisms operate across the BMI range (from underweight to obesity) and if they play a role in childhood.

In our study, emotional overeating was bi-directionally associated with BMI across childhood. Remarkably, emotional overeating was not cross-sectionally associated with BMI at 4 years, and there was only a low correlation of emotional overeating over time. This suggests that variation in this trait is expressed with increasing age, probably due to more free access to foods, although it might also reflect a later recognition of parents. In line with this, Ashcroft et al. (2008) also reported an increase in emotional overeating from age 4 years to 11 years [41]. Furthermore, a British twin study showed that emotional overeating was explained mostly by environmental variance, rather than genetic influences. This suggests that overeating in response to negative emotions is a learned behavior, which might explain the increase in variation over time [42]. These findings suggest that a vicious cycle may appear with emotional eating resulting in more food intake and weight gain, while in turn, excess weight may lead to overeating, e.g. due to higher body dissatisfaction [43]. This implies that targeting emotional overeating during childhood might be useful for obesity prevention.

### Strengths and weaknesses

Strengths of this study were its prospective design including repeated measurements of eating behavior, BMI and body composition, and a large sample size. However, there are also some limitations. First, in-depth body composition measures of fat mass and fat-free mass were not available at the age of 4 years. This limits our conclusions regarding directionality between body composition and eating behavior. Second, no information on eating behavior was available before 4 years of age, while more food approaching and less food avoidant eating behaviors in infancy may precede a higher BMI at 4 years. Thirdly, eating behavior was only assessed using mother-reported questionnaires. Reports on child eating behavior can be sensitive to reporter-bias. However, we tried to minimize this bias by adjusting for several maternal characteristics in our analyses. While these covariates included in our analyses had only a minimal effect on the results, it cannot be completely ruled out that mother’s attitudes affected her ratings. On the other hand, a validation study showed that parent-reported food responsiveness, satiety responsiveness and enjoyment of food were associated with a range of behavioral food intake tests, such as total energy intake and eating rates [27]. Emotional overeating was not examined in that study. Finally, generalizability may be limited, because the non-response analyses indicated that participants who were lost to follow-up in the Generation R study were more often from a non-Dutch background, lower household income and higher maternal BMI, which are risk factors for a higher BMI [44, 45].

## Conclusion

Although it is often assumed that unhealthy eating behavior is a risk factor for childhood obesity, our results do not support this. Instead, we found evidence for the reverse direction: a higher BMI –and particularly a higher fat mass - at pre-school age predicted more food approaching and less food avoidant behaviors at the age of 10 years. These findings suggest that children who are already on a high BMI trajectory at a young age develop more excess weight through an up-regulation in appetite. Thus, it may not be possible to prevent childhood obesity by targeting eating behaviors in middle childhood. Future studies covering infancy to adolescence are needed to replicate and explain our novel findings.

## Additional file

Additional file 1: **Figure S1.** Flowchart of the study sample. (DOCX 27 kb)

## Abbreviations

BMI: Body Mass Index
BSI: Brief Symptom Inventory
CEBQ: Child Eating Behavior Questionnaire
CI: Confidence Interval
DXA: Dual-energy-X-ray-Absorptiometry
FFMI: Fat Free Mass Index
FMI: Fat Mass Index
