# Supplementary material for: Eating behavior and body composition across childhood: a prospective cohort study
Source: Int J Behav Nutr Phys Act. 2018 Oct 1;15:96. doi: 10.1186/s12966-018-0725-x (PMC6167809; doi:10.1186/s12966-018-0725-x)
Supplement: Supplementary file 1 — Figure S1. Flowchart of the study sample. (DOCX 27 kb) [file 12966_2018_725_MOESM1_ESM.docx]

**Additional file 1: Figure S1. Flowchart of the study sample**

**N=7294**
Children with consent for participation in the postnatal phase until 10 years.

**N=2814**
No information on eating behavior at the age of 4 years

**N=4480**

Children with information on eating behavior at 4 years.

**N=966**
No information on eating behavior at the age of 10 years

**N=3514**
Children with information on eating behavior at 10 years.

**N=183**
No information on BMI or body composition at the age of 10 years

**N=3331**
Children with full information on eating behavior at 4 and 10 years , and BMI and body composition at 10 years.

N=2195 also had BMI available at 4 years (missing data imputed)
